# Treatment of IgA Nephropathy: A Rapidly Evolving Field

**DOI:** 10.1681/ASN.0000000000000242

**Published:** 2023-09-29

**Authors:** Khalil El Karoui, Fernando C. Fervenza, An S. De Vriese

**Affiliations:** 1 Department of Nephrology, Hôpital Tenon, Sorbonne Université, Paris, France; 2 Division of Nephrology and Hypertension, Mayo Clinic, Rochester, Minnesota; 3 Division of Nephrology and Infectious Diseases, AZ Sint-Jan Brugge, Brugge, Belgium; 4 Department of Internal Medicine, Ghent University, Ghent, Belgium

**Keywords:** IgA nephropathy, immunosuppression, primary GN, proteinuria, glomerular diseases

## Abstract

The pivotal event in the pathophysiology of IgA nephropathy is the binding of circulating IgA-containing immune complexes to mesangial cells, with secondary glomerular and tubulointerstitial inflammation and fibrosis. The paramount difficulty in the management of IgA nephropathy is the heterogeneity in its clinical presentation and prognosis, requiring an individualized treatment approach. Goal-directed supportive care remains the bedrock of therapy for all patients, regardless of risk of progression. Sodium–glucose transporter 2 inhibitors and sparsentan should be integral to contemporary supportive care, particularly in patients with chronic kidney damage. Pending the development of reliable biomarkers, it remains a challenge to identify patients prone to progression due to active disease and most likely to derive a net benefit from immunosuppression. The use of clinical parameters, including the degree of proteinuria, the presence of persistent microscopic hematuria, and the rate of eGFR loss, combined with the mesangial hypercellularity, endocapillary hypercellularity, segmental glomerulosclerosis, tubular atrophy/interstitial fibrosis, crescents score, is currently the best approach. Systemic glucocorticoids are indicated in high-risk patients, but the beneficial effects wane after withdrawal and come at the price of substantial treatment-associated toxicity. Therapies with direct effect on disease pathogenesis are increasingly becoming available. While targeted-release budesonide has garnered the most attention, anti–B-cell strategies and selective complement inhibition will most likely prove their added value. We propose a comprehensive approach that tackles the different targets in the pathophysiology of IgA nephropathy according to their relevance in the individual patient.

## Introduction

IgA nephropathy, histologically defined by dominant or codominant IgA mesangial deposits, is the most prevalent primary GN worldwide.^1^ The clinico-biologic spectrum ranges from asymptomatic hematuria to rapidly progressive GN. Cohort studies from the United Kingdom,^2^ China,^3^ and Japan^4^ reveal a significant heterogeneity in disease course, with a substantial proportion of patients progressing to kidney failure over the course of decades. IgA nephropathy is more prevalent and has a more adverse prognosis in Asian patients,^5^ which cannot be entirely explained by biopsy policies,^6^ because Asian ethnicity is associated with a higher rate of kidney failure even in European or North American studies.^2,7^

**Figure 1:**
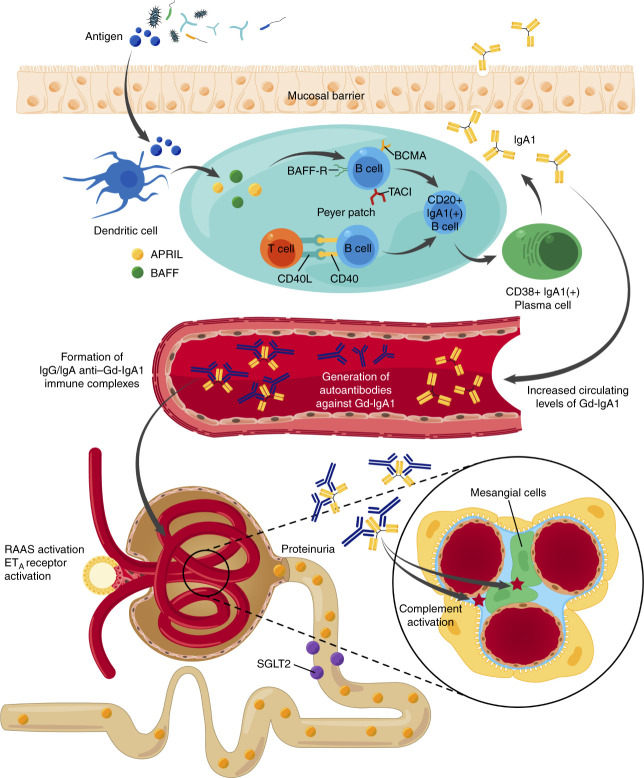
**Pathogenesis of IgA nephropathy: the four-hit model.** Mucosal IgA is produced within the MALT, more particularly in the GALT, including the Peyer patches, and the NALT, where it plays a key role in the host defense against pathogens. Antigens from the gastrointestinal and respiratory tract are processed by the innate immune system, among which dendritic cells. Class switching of naïve B cells to IgA1(+) B cells occurs *via* T-cell–dependent (including CD40–CD40L interaction) and T-cell–independent mechanisms, the latter with a critical role for BAFF and APRIL. Both cytokines stimulate B cells *via* TACI, BCMA, or BAFF-R. IgA1(+) B cells differentiate into IgA1(+) B plasma cells that traffic toward the mucosal surface and produce IgA1, which subsequently enters the lumen. In IgA nephropathy, genetic defects in the enzymes responsible for the galactosylation of IgA1 lead to the formation of Gd-IgA1. The first hit in the pathogenesis of IgA nephropathy is the systemic accumulation of Gd-IgA1, thought to be secreted by gut or respiratory tract-homing Gd-IgA1(+) B cells with spillover from mucosal sites or from B cells that have mishomed to systemic sites.^9^ The finding of increased circulating levels of intestinal-activated Gd-IgA1(+) B lymphocytes and Gd-IgA1(+) plasma cells^10,11^ also supports this hypothesis. The second hit is the development of autoantibodies directed against the poorly galactosylated region of IgA1. Subsequent circulating immune complex formation consisting of Gd-IgA1 and anti–Gd-IgA1-IgG, IgA, and/or IgM antibodies represents the third hit. The fourth hit entails binding of these immune complexes to mesangial cells, leading to mesangial cell activation. This sets in motion a number of proinflammatory and profibrotic pathways, amplified by complement, RAAS, and ET_A_ activation. The ultimate result is progressive glomerular and tubulointerstitial injury. APRIL, a proliferation-inducing ligand; BAFF, B-cell activating factor; BAFF-R, BAFF receptor; BCMA, B-cell maturation antigen; ET_A_, endothelin receptor type A; GALT, gut-associated lymphoid tissue; Gd-IgA1, galactose-deficient IgA1; MALT, mucosa-associated lymphoid tissue; NALT, nasopharynx-associated lymphoid tissue; TACI, transmembrane activator and calcium-modulating ligand (CAML) interactor; RAAS, renin–angiotensin–aldosterone system.

**Figure 2:**
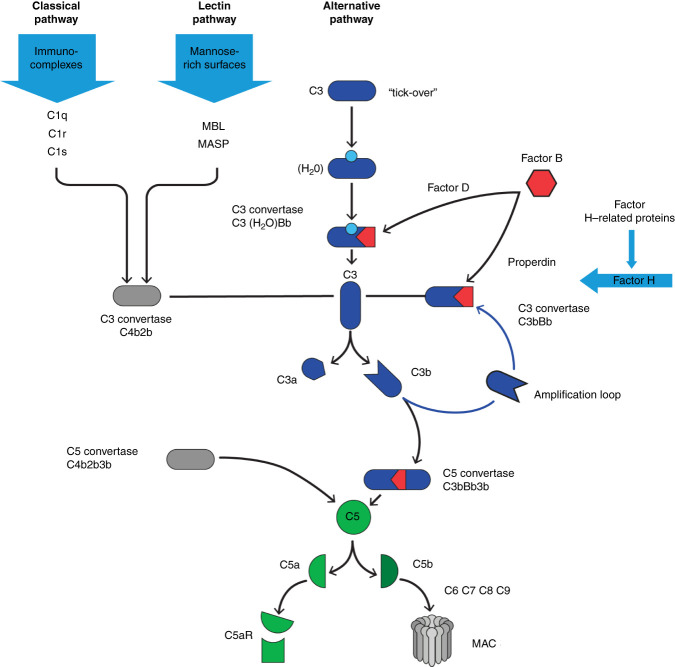
**Involvement of the complement pathway in IgA nephropathy.** The complement system can be activated by the classical, lectin, and alternative pathways, all resulting in the formation of C3 convertases. The classical pathway is initiated by immune complexes that interact with C1q. The lectin pathway is activated by the binding of MBLs and MASP to carbohydrate moieties found primarily on the surface of microbial pathogens. The alternative pathway is capable of autoactivation by a mechanism called “tick-over” of C3. Any of the C3 convertases can cleave C3 to C3a and C3b, producing more C3 convertase in a powerful amplification loop and fully activating the complement system. The terminal complement cascade is initiated by the C5 convertase and ultimately generates the MAC complex. Factor B is the proteolytically active component of the C3 and C5 convertases. The plasma protein properdin stabilizes C3bBb. C3a and C5a are strong anaphylatoxins. Factor H is an important negative regulator of the alternative pathway. Fine-tuning occurs through the factor H–related proteins that compete with factor H and thus prevent deactivation of C3b. In patients with IgA nephropathy, components of the alternative (properdin, factor H, factor H-related proteins and factor B) and lectin (C4d in the absence of C1q, MBL, MASP) pathways are found in the mesangial deposits and correlate with prognosis.^17^ The association of circulating factor H–related proteins and disease prevalence/progression,^18,19^ the frequent observation of thrombotic microangiopathy lesions in IgA nephropathy relative to other forms of immune-mediated GN,^20–22^ and the response of severe forms of IgA nephropathy to C5 inhibition^23^ further support a role for complement dysregulation in IgA nephropathy. MAC, membrane attack complex; MASP, mannose-binding lectin-associated serine protease; MBLs, mannose-binding lectins.

IgA nephropathy is believed to result from a succession of several pathogenetic hits^8^ (Figure 1). The first hit is represented by increased circulating levels of galactose-deficient IgA1 (Gd-IgA1). Gd-IgA1 is recognized as self-antigen and forms nephritogenic immune complexes with anti–Gd-IgA1-IgG, IgA, and/or IgM.^12^ Subsequent deposition of these circulating IgA-containing immune complexes in the glomerular mesangium instigates several injury pathways, resulting in glomerular inflammation and fibrosis. The contribution of specific receptors for IgA-containing immune complexes in mesangial cells remains controversial.^13–16^ The pivotal role of the complement system, particularly the lectin and alternative pathways, in mediating glomerular inflammation is well recognized^17^ (Figure 2). Increased intraglomerular pressure after nephron reduction and tubulointerstitial toxicity of proteinuria further favors nephron loss and progression of CKD. The intricate and multifaceted pathophysiology of IgA nephropathy implies that solely targeting one factor with treatment will not be enough. Instead, a comprehensive approach that tackles the various components is necessary.

## Nonimmunologic Treatment

### Renin–Angiotensin–Aldosterone Inhibition

The well-known beneficial effects of renin–angiotensin–aldosterone inhibition (RAASi) in proteinuric kidney diseases are mediated by a lowering of BP and intraglomerular hypertension, with a reduction of proteinuria and downstream glomerular injury, independently of the specific pathophysiology of the underlying kidney disease.^24,25^

A few randomized controlled trials (RCTs) have documented the nephroprotective effects of angiotensin-converting enzyme inhibitors,^26,27^ angiotensin II receptor blockers,^28^ and dual RAASi,^29^ specifically in IgA nephropathy patients with hypertension and significant proteinuria. No specific data exist for patients with proteinuria <0.5 g/d and normal BP. Importantly, a substantial number of patients with IgA nephropathy develop aldosterone breakthrough after long-term RAASi with documented loss of clinical efficacy.^30^ Consequently, steroidal (spironolactone) and nonsteroidal (finerenone) mineralocorticoid receptor antagonists may have an additive benefit in IgA nephropathy, as demonstrated for diabetic kidney disease,^31^ but trials dedicated to IgA nephropathy have not been conducted.

### Sodium–Glucose Transporter 2 Inhibition

The nephroprotective effects of sodium–glucose transporter 2 (SGLT2) inhibitors beyond their ability to lower glycemia and BP have been attributed to tubuloglomerular feedback-induced afferent arteriolar vasoconstriction and augmented proximal tubular pressure, both conducive to decreased glomerular capillary pressure, and to reduced renal oxygen consumption.^32^ Moreover, SGLT2 inhibition may have direct protective effects on podocytes.^33^

The Dapagliflozin and Prevention of Adverse outcomes in Chronic Kidney Disease (DAPA-CKD)^34^ and Study of Heart and Kidney Protection With Empagliflozin^35^ trials unequivocally demonstrated the nephroprotective effects of SGLT2 inhibition in albuminuric nondiabetic CKD, even in patients with eGFR <30 ml/min per 1.73 m^2^. Both trials recruited a large number of participants with IgA nephropathy (270 and 817, respectively), excluding those on recent immunotherapy. They did not require a standardized run-in phase to optimize supportive care, although many patients received RAASi at enrollment. In a prespecified *post hoc* analysis of the IgA cohort of the DAPA-CKD,^36^ dapagliflozin reduced the risk of the primary composite outcome (sustained >50% decline in eGFR, ESKD, or death from a kidney disease-related or cardiovascular cause) by 71% after a median follow-up of 2.1 years, with similar effects across prespecified subgroups according to baseline eGFR and proteinuria. Judging from the baseline clinical characteristics and the exclusion criterium of recent treatment with immunosuppressive agents, the DAPA-CKD trial preferentially recruited older patients with IgA nephropathy in a more chronic phase of the disease,^37^ consistent with their anticipated role as long-term nephroprotective agents independent of the specific pathophysiology of IgA nephropathy.

### Endothelin Receptor Antagonism

Sparsentan is a nonimmunosuppressive selective endothelin type A receptor and angiotensin II subtype 1 receptor antagonist. The rationale for this dual inhibition is supported by studies in experimental CKD showing that combined antagonism has better hemodynamic, anti-inflammatory, antifibrotic, and podocyte protective effects than either molecule alone.^38^

The Study of the Effect and Safety of Sparsentan in the Treatment of Patients With IgA Nephropathy trial^39^ randomized 404 IgA nephropathy patients with persistent proteinuria (≥1 g/d) despite full-dose RAASi to either sparsentan or irbesartan. Interim analysis after the 9-month follow-up showed that sparsentan produced a meaningful reduction in proteinuria (between-group relative reduction of 41%). Differences in BP between the treatment arms were minimal, suggesting that the proteinuria-lowering effect of sparsentan is partly independent from the BP-lowering effect. Treatment-emergent adverse events were similar between both groups. The promising preliminary results led to the conditional accelerated approval of sparsentan by the US Food and Drug Administration for adults with IgA nephropathy at risk of rapid disease progression, generally with UPCR ≥1.5 g/g.^40^ Sparsentan is expected to be priced 9900$ per month in the United States.^41^ The final analysis will assess kidney function outcomes after 2 years of treatment.

## Broad-Acting Immunosuppressants

### Systemic Glucocorticoids

Systemic glucocorticoids have potent effects across the spectrum of immune function such that suppression of Gd-IgA1 production and subsequent immune complex formation and glomerular inflammation could be expected. However, one study found a reduction of IgA1 plasma cells but not of Gd-IgA1 plasmablasts/plasma cells in patients with IgA nephropathy treated with prednisone,^10^ suggesting that systemic glucocorticoids do not eliminate the trigger of the disease. Glucocorticoids may also directly affect podocyte and parietal epithelial cell homeostasis.^42,43^

Glucocorticoids have been used for decades in patients with IgA nephropathy considered to be at high risk of disease progression. A Cochrane review conducted in 2020 found that in patients with proteinuria >1 g/d, a course of glucocorticoids lowered urinary protein excretion, induced more complete remission, and reduced the risk of progression to kidney failure compared with placebo or standard of care.^44^ Many of the historical studies included in this analysis have been criticized for not optimizing supportive care and RAASi during a standardized run-in phase. This shortcoming was appropriately addressed in the STOP-IgA nephropathy^37^ and TESTING^46^ trials, both of which recruited patients with significant residual proteinuria (>0.75 g/d in STOP-IgA nephropathy and ≥1 g/d in TESTING) despite optimal conservative treatment. Although they are often framed as yielding opposite conclusions, the core message from both trials is basically similar. TESTING found that glucocorticoids significantly reduced the frequency of the composite end point (40% decrease in eGFR or ESKD or renal-related death, observed in 28.8% of the glucocorticoid arm versus in 43.1% of the placebo arm), but differences in protein excretion were not sustained, and after an early eGFR increase in the glucocorticoid group, subsequent eGFR decline occurred at the same rate as in the placebo group. In the STOP-IgA nephropathy trial, an initial reduction of proteinuria and higher proportion of full remission was observed in the glucocorticoid group, but this difference disappeared at 36 months and did not translate into a significant effect on the annual decline of eGFR. Although the disparities between the trials have often been attributed to differences in ethnicity (White in STOP-IgA nephropathy and Asian in TESTING), they may rather boil down to the risk profile of the patients. Asian people are known to have more aggressive disease that may be more amenable to immunosuppression, thus improving the power of the study. The participants of STOP-IgA nephropathy may have had a lower baseline risk for progression, as illustrated by the slow eGFR decline in the control group, thus hampering the ability of the trial to reveal a significant benefit of a single course of glucocorticoids. An updated meta-analysis incorporating both trials confirms the early effectiveness of systemic glucocorticoids, regardless of race, glucocorticoid regimen, and background therapy.^47^

Glucocorticoid toxicity did not seem to be an issue in the older trials, possibly due to underreporting of serious adverse events. By contrast, TESTING and STOP-IgA nephropathy revealed substantial treatment-associated toxicity, including infections requiring hospitalization, diabetes, and death due to sepsis.^45,46^ The reduced dose regimen in TESTING maintained its efficacy while improving its tolerability but still resulted in significant adverse events.^46^ Remarkably, almost half of the severe infections requiring hospitalization were caused by *Pneumocystis jirovecii*, *Nocardia*, and *Cryptococcus*,^46^ prompting the investigators to add *Pneumocystis* prophylaxis in the reduced dose cohort. Interestingly, two genetic risk loci for IgA nephropathy (CARD9 and VAV3) play a role in the defense against these organisms,^48^ providing a rationale for the increased infection risk in patients with IgA nephropathy and supporting the routine use of antibiotic prophylaxis during intensive immunosuppression.

Taken together, systemic glucocorticoids seemingly reduce renal inflammation with beneficial effects as long as therapy is continued, but benefits wane after withdrawal, presumably because the underlying pathophysiologic process is not fundamentally addressed. Although repeated glucocorticoid courses may be a solution in selected patients with relapsing-remitting disease, long-term glucocorticoid use is undesirable in view of the well-documented adverse effects.

### Mycophenolate Mofetil

Mycophenolate mofetil (MMF) has potent and relatively selective suppressive effects on activated T and B lymphocytes. In addition, MMF prevented renal inflammation, glomerulosclerosis, and tubulointerstitial injury in the remnant kidney, suggesting that it may also attenuate nonimmune renal injury.^49^ Historical studies of MMF in monotherapy or in combination with glucocorticoids, conducted mainly in patients with advanced IgA nephropathy, have yielded conflicting results.^8^ The more recent Effect of Mycophenolate Mofetil on Renal Outcomes in Advanced Immunoglobulin A Nephropathy trial, performed in patients with proteinuria >1 g/d, hematuria, and eGFR between 30 and 60 ml/min per 1.73 m^2^ or with persistent hypertension, revealed that 18-month MMF reduced the risk of a composite end point of doubling of serum creatinine, ESKD, or death due to kidney or cardiovascular cause by 77% after 3 years of follow-up.^50^ Interestingly, pathologic findings at presentation (41% C1, 55% T2, 62% glomerulosclerosis >50%) suggested already advanced disease. Furthermore, subgroup analyses showed that patients with eGFR 30–50 ml/min per 1.73 m^2^ benefited equally or more from MMF compared with those with eGFR >50 ml/min per 1.73 m^2^. These observations suggest that even in the presence of sclerotic and fibrotic changes, active immunologic disease (as manifested by the presence of persistent hematuria in 100% of the patients) amenable to immunosuppression may still be present and argue against a defeatist approach in the face of kidney failure. Alternatively, mitigation of nonimmune renal injury by MMF may have played a role.^49^ In the post-trial phase, urinary protein excretion increased and the annual rate of eGFR decline accelerated after discontinuation of MMF, indicating that the beneficial effect of MMF does not last after withdrawal. In patients with active proliferative lesions (cellular and fibrocellular crescents, endocapillary hypercellularity, or necrosis), MMF combined with low-dose glucocorticoids was equally effective and resulted in fewer side effects than high-dose glucocorticoids,^51^ providing a valuable alternative for patients unable to support high-dose glucocorticoids. In a large retrospective cohort of 3946 patients with IgA nephropathy, new users of immunosuppressive agents had a 40% lower risk of the primary outcome (a composite of 40% eGFR decline, ESKD, and all-cause mortality) and more serious adverse events than propensity score-matched recipients of supportive care.^52^ The effect size was comparable for glucocorticoid monotherapy, MMF monotherapy, or the combination of both.^52^

## Therapies that Target the Formation of Gd-IgA1 and Anti–Gd-IgA1 Antibody

### Targeted-Release Budesonide

Targeted-release formulation (TRF)–budesonide is packaged in a pH-sensitive starch capsule such that approximately 70% of the active compound is released in the distal ileum and proximal colon and delivered to the Peyer patches, where most of the synthesis of IgA and Gd-IgA1 takes place. Because of first-pass metabolism in the liver, <10% of the active compound enters the systemic circulation.^53^ TRF-budesonide is postulated to selectively affect the immune cells in the gut, which may translate into reduced the levels of secretory IgA, circulating Gd-IgA1, B-cell activating factor (BAFF), B-cell maturation antigen, transmembrane activator and calcium modulating ligand interactor, and circulating IgA-IgG immune complexes^54^ as well as downstream proinflammatory and fibrotic pathways.^55^

The phase 2 The Effect of Nefeconin Patients With Primary IgA Nephropathy at Risk of Developing End-stage Renal Disease (NEFIGAN)^56^ and phase 3 NefIgArd^57^ studies recruited patients with persistent proteinuria (≥1 g/d) despite optimized RAASi. A 9-month course of TRF-budesonide resulted in a significant reduction of proteinuria^56,57^ and preservation of eGFR^57^ compared with placebo, leading to its conditional accelerated approval in the United States and European Union for adult patients with primary IgA nephropathy at risk of rapid disease progression with UPCR ≥1.5 g/g.^58,59^ The recently published results of the extension study revealed a sustained benefit on eGFR and proteinuria after 2 years,^60^ but proteinuria started to increase again after 12 months, and the eGFR decline in the TRF-budesonide arm ran in parallel with that of the placebo arm, suggesting that—similarly to oral glucocorticoids—the favorable effects of TRF-budesonide wane over time. Treatment-related side effects were mild, although signs of systemic corticosteroid exposure were noted in up to 41% of patients in the NEFIGAN study.^56^

A major constraint to the widespread use of TRF-budesonide is its economic cost ($14,160 for 1 month of treatment^61^). It should be noted that the postulated selective effect of TRF-budesonide on the Peyer patches has not been directly demonstrated. Peyer patches are concentrated near the ileocecal junction, but with a large interindividual variation in distribution.^62^ In the absence of a direct comparison with other enteric-coated budesonide preparations developed for the treatment of inflammatory bowel disease, the added value of TRF-budesonide compared with the much cheaper traditional budesonide formulations is speculative.^63,64^

### B-Cell and Plasma Cell–Targeted treatment

In a RCT of 34 patients with IgA nephropathy, rituximab failed to affect proteinuria, kidney function, serum levels of Gd-IgA1, or antibodies against Gd-IgA1, despite adequate depletion of CD20(+) B cells.^65^ These results imply that the cells pivotal for Gd-IgA1 and anti–Gd-IgA1 antibody formation may be CD20(−) and thus unaffected by rituximab.

Patients with IgA nephropathy have increased the levels of CD38(+) B cells and plasma cells,^66^ which are believed to be responsible for the increased Gd-IgA1 and anti–Gd-IgA1 antibody production. Felzartamab, a recombinant fully human monoclonal antibody against CD38, is currently in a phase 2a trial for patients with IgA nephropathy (IGNAZ; NCT05065970) (Table 1).

**Table 1:** Molecules in clinical development for the treatment of IgA nephropathy

Treatment		Target	Phase	Identifier	Outcome	Estimated Study Completion Date
Supportive care						
SGLT2i	CLIgAN	SGLT2		NCT04662723	UPE	December 26
Sparsentan	SPARTAN	ERA+ARB		NCT04663204	24 h-UPCR and eGFR	November 23
Sparsentan	SPARTACUS	ERA+ARB	II	NCT05856760	UACR (sample)	December 24
Sparsentan	PROTECT	ERA+ARB	III	NCT03762850	24 h-UPCR	July 26
Sparsentan (pediatrics)	EPPIK	ERA+ARB	II	NCT05003986	UPCR	June 25
Atrasentan	ALIGN	ERA+ARB		NCT04573478	UPCR	December 25
Atrasentan	ASSIST	ERA+ARB		NCT05834738	UPCR	October 25
Atrasentan	AFFINITY	ERA+ARB	II	NCT04573920	UPCR	February 26
SC 0062		ERA	II	NCT05687890	UACR	April 25
Steroids						
Steroids		Systemic	III	NCT03468972	eGFR	May 23
Steroids	CLIgAN	Systemic	III	NCT04662723	UPE	December 26
Steroids	TIGER	Systemic	III	NCT03188887	UPCR (sample)+eGFR	January 24
Steroids		Systemic	III	NCT04833374	24 h-UPE	December 23
B and plasma cell						
Rituximab		CD20	IV	NCT05824390	UPE	October 23
Rituximab	RITA	CD20	IV	NCT04525729	UPE	December 23
Feltarzamab	IGNAZ	CD38	II	NCT05065970	UPE	May 24
Belimumab	BELIGA	BAFF	II	EudraCT: 2017–004366-10	UPE	
Sibeprenlimab	enVISion	APRIL	II	NCT04287985	24 h-UPCR	June 23
Sibeprenlimab	VISIONARY	APRIL	III	NCT05248646	24 h-UPCR	December 26
Ataticept	ORIGIN-3	BAFF+APRIL	II/III	NCT04716231	24 h-UPCR	July 2028
Telitaticept		BAFF+APRIL	II	NCT04905212	24 h-UPE	January 24
Bortezomib		Proteasome	II	NCT05383547	24 h-UPE	December 23
AT-1501		CD40 L	II	NCT05125068	24 h-UPCR	August 25
Complement						
Iptacopan	APPLAUSE-IgA nephropathy	CF B		NCT04578834	24 h-UPCR+eGFR	October 25
Narsoplimab		MASP-2	III	NCT03608033	24 h-UPE	April 23
Vermicopan		CF D	II	NCT05097989	24 h-UPE	August 26
Pegcetacoplan		C3	II	NCT03453619	UPCR	December 23
Ravulizumab	SANCTUARY	C5	II	NCT04564339	24 h-UPE	June 25
Cemdisiran		C5 RNA	II	NCT03841448	24 h-UPCR	February 25
IONIS-FB-LRx		CF B RNA	II	NCT04014335	24 h-UPE	December 23
RO7434656	IMAGINATION	CF B RNA	III	NCT05797610	24 h-UPCR	September 30
KP 104		C3 convertase+C5	II	NCT05517980	24 h-UPCR	September 25
Microbiome						
Enterobacteriaceae capsules		Microbiome	II	NCT05182775	24 h-UPE	December 23

SGLT2, sodium–glucose transporter 2; CLIgAN, Multicentre Clinical Study to Evaluate the Effect of Personalized Therapy on Patients With Immunoglobulin A Nephropathy; UPE, urinary protein excretion; EPPIK, Study of Sparsentan Treatment in Pediatrics With Proteinuric Glomerular Diseases; ERA, Endothelin Receptor Antagonism; ARB, angiotensin II receptor blocker; UPCR, urinary protein-to-creatinine ratio; UACR, urinary albumin-to-creatinine ratio; PROTECT, A Study of the Effect and Safety of Sparsentan in the Treatment of Patients With IgA Nephropathy; BAFF, B-cell activating factor; APRIL, a proliferation-inducing ligand; MASP, mannose-binding lectin-associated serine protease.

Several lines of evidence support a pathogenetic role for BAFF and a proliferation-inducing ligand (APRIL) in the pathogenesis of IgA nephropathy and have provided a rationale for therapies that specifically target these cytokines (Table 1).^67^ Atacicept, a fusion protein that can bind both BAFF and APRIL, was evaluated in a phase 2 trial of 116 proteinuric IgA nephropathy patients. Atacicept 150 mg reduced serum Gd-IgA1 by 60% and decreased proteinuria by 33% at week 24 (difference versus placebo=28%, *P* = 0.047).^68^

Bortezomib, a proteasome inhibitor targeting plasma cells, had mixed effects in eight IgA nephropathy patients treated with four doses (remission of proteinuria in three and no effects in four) after the 1-year follow-up.^69^

## Therapies that Target Complement-Mediated Inflammation

### Avacopan

Avacopan is an oral C5a receptor inhibitor. In a pilot study of seven patients with IgA nephropathy and proteinuria >1 g/d despite RAASi, 12 weeks of avacopan led to a reduction of proteinuria, that persisted at 24 weeks.^70^

### Iptacopan

Iptacopan is an oral selective factor B inhibitor that thwarts the amplification of the initial complement response through the alternative pathway, thus preventing overactivation of the complement system. However, it does not inhibit direct activation of the classical and lectin pathway, explaining the absence of serious infectious complications in the reports so far. In a phase 2 study of 112 patients with IgA nephropathy, iptacopan (200 mg twice daily) reduced proteinuria by up to 40% after 6 months.^71^ The phase 3 APPLAUSE-IgA nephropathy (NCT04578834) is currently ongoing and aims to recruit 450 patients (Table 1).

### Narsoplimab

Narsoplimab is a humanized monoclonal antibody against mannose-binding lectin-associated serine protease-2 that selectively inhibits the lectin pathway. In a phase 2 study of 12 high-risk patients with IgA nephropathy, proteinuria at 18 weeks was not different in the narsoplimab and vehicle arms. However, longer exposure to narsoplimab in an open phase extension revealed a 61% reduction of proteinuria after 31–54 weeks.^72^ A phase 3 trial (NCT03608033) is presently recruiting (Table 1).

## Hydroxychloroquine

Hydroxychloroquine has multiple mild effects on the immune system, including the reduction of proinflammatory cytokine production, activation of dendritic cells, and proliferation of T and B cells, many of which may potentially contribute to a beneficial effect in IgA nephropathy. Several retrospective studies reported proteinuria reduction in patients with IgA nephropathy treated with hydroxychloroquine.^73–76^ In a RCT of 60 IgA nephropathy patients on optimized standard of care, 6 months of hydroxychloroquine decreased proteinuria by 48% as compared with a 10% increase in the placebo arm.^77^ Importantly, hydroxychloroquine has a well-described favorable safety profile even after prolonged exposure,^78^ suggesting that it could be proposed on a long-term basis.

## Proposal for a Therapeutic Strategy

### Risk Stratification

A broad range of clinical parameters, histopathologic data, and other biomarkers have been researched in an attempt to identify patients prone to disease progression. However, an adverse renal prognosis does not necessarily imply a high probability of response to immunosuppressive therapy. The key element in adequate risk stratification is therefore not only to predict which patients have progressive kidney disease but also to differentiate patients with active inflammatory disease from those with predominantly chronic damage.

#### Clinical Parameters

The severity of proteinuria on presentation has been consistently shown to be a risk factor for progressive kidney function loss.^79^ The rate of progression is low when proteinuria is <1 g/d and is greatest when it is >3–3.5 g/d. In addition, remission of proteinuria is associated with improved kidney outcomes,^80,81^ supporting the notion that every effort should be made to reduce proteinuria to <1 g/d. The Validation Study of the Oxford Classification of IgAN (VALIGA) study revealed a linear correlation between baseline proteinuria and response to glucocorticoids, with the most pronounced effect noted when proteinuria was ≥3 g/d.^82^ However, the STOP-IgA nephropathy^37^ and The Therapeutic Effects of Steroids in IgA Nephropathy Global (TESTING)^46^ trials found no difference in response to immunosuppression with respect to baseline proteinuria. This discrepancy may be explained by the fact that proteinuria does not inherently indicate active disease but may result from glomerular sclerosis and tubular damage. Conversely, a substantial proportion of patients with proteinuria <1 g/d but with high-risk histologic features and significant microscopic hematuria still develop progressive loss of kidney function,^83^ indicating that proteinuria <1 g/d by itself does not guarantee a favorable outcome.^2^

Microscopic hematuria results from glomerular capillary wall damage caused by immune complex deposition and is therefore a *prima facie* sign of glomerular inflammation. Although historical studies of the prognostic value of hematuria have yielded conflicting results, more recent studies with longitudinal follow-up show that pronounced and persistent hematuria is associated with an adverse renal prognosis,^84,85^ while remission of hematuria results in a slower decline of renal function.^85^ Persistent microscopic hematuria thus has emerged as a biomarker of disease activity in IgA nephropathy, independent of proteinuria, but even more so in the presence of proteinuria.^86^

#### Histologic Data

Each of the components of the revised Oxford classification of IgA nephropathy M=mesangial hypercellularity, E=endocapillary hypercellularity, S=segmental glomerulosclerosis, T=tubular atrophy/interstitial fibrosis, C=crescents (MEST-C) score has been shown to individually predict renal outcome, independent of clinical data.^87,88^ A small disclaimer has to be made for endocapillary proliferation, the presence of which was not associated with kidney failure in a meta-analysis of retrospective data.^88^ However, its negative predictive value may have been overruled by a greater use of immunosuppressive therapy in this group. Indeed, when patients treated with immunosuppression were specifically excluded, a strong relation between endocapillary proliferation and adverse outcome was found.^89^

Mesangial proliferation, endocapillary proliferation, and crescents are active inflammatory lesions, with the potential to identify high-risk patients who would benefit from immunosuppressive treatment. M1, E1, and C1 lesions have been shown to be sensitive to glucocorticoids and MMF in retrospective studies.^90–94^ In a prospective study, 6 months of glucocorticoids reduced E, S, and C lesions.^51^ However, TESTING^46^ and a secondary analysis of a limited number of biopsies (*n*=70) from Supportive Versus Immunosuppressive Therapy for the Treatment of Progressive IgA Nephropathy [STOP-IgA] nephropathy^95^ found no difference in outcome between glucocorticoids and placebo for those with or without endocapillary^46^ or mesangial hypercellularity,^46,95^ although the substantial delay between kidney biopsy and trial enrollment (5 months in TESTING and 6–92 months in STOP-IgA nephropathy) should be taken into account.

Conversely, segmental glomerulosclerosis and tubular atrophy/interstitial fibrosis are markers of chronic damage, suggesting that immunosuppression should be avoided in patients who exclusively have these lesions. T2 lesions have indeed been associated with the absence of response to glucocorticoids in both Asian and Caucasian cohorts.^92,93^ The therapeutic responsiveness of S1 lesions is more controversial. A proportion of patients with S1 lesions respond clinically and pathologically to glucocorticoids,^93,96–98^ supporting the existence of a specific form of podocytopathy in some IgA nephropathy patients. These observations have led to an updated recommendation of the Oxford classification to subclassify S1 lesions according to the presence of signs of podocyte damage, such as podocyte hypertrophy or tip lesions.^87^

The ability of histologic markers to predict a benefit of early glucocorticoids on top of RAASi versus RAASi alone is the subject of two ongoing prospective studies in European IgA nephropathy patients. The Multicentre Clinical Study to Evaluate the Effect of Personalized Therapy on Patients With Immunoglobulin A Nephropathy (CLIgAN) (NCT04662723) studies a subgroup with E1 and/or C1 lesions, while the treatment of IgA nephropathy according to renal lesions (TIGER, NCT03188887) recruits patients with MEST-C >1 (excluding T2).

#### International IgA Nephropathy Prediction Tool

The International IgA Nephropathy Prediction Tool,^99^ freely available at www.qxmd.com, calculates the 5-year risk of a 50% decrease in eGFR or development of ESKD, based on a number of clinical (eGFR, BP, proteinuria, age, race/ethnicity, use of RAASi) and histologic (MEST score) variables at the time of kidney biopsy. The presence or absence of hematuria and of glomerular crescents is not included in the prediction formula. Because several variables, particularly BP and use of RAASi, may change substantially after kidney biopsy, the tool was refined to provide a risk estimate at 1 and 2 years after kidney biopsy.^100^ Application of the prediction tool to patients from the STOP-IgA nephropathy trial revealed a significant overlap in risk estimates between patients who had or had not reached the primary composite end point of either 50% eGFR decrease or ESKD.^101^ The tool was endorsed by the 2021 Kidney Disease Improving Global Outcomes guidelines to inform patients about their risk of progression, but not to guide the decision to use immunosuppression.^102^

#### Other Biomarkers

A high intensity of C3 deposition^103,104^ and the presence of C4d deposition^105,106^ in the kidney biopsy correlated with an unfavorable clinical outcome, highlighting the importance of complement-induced inflammation. A higher versus lower intensity of CD206^+^ and CD68^+^ macrophage infiltration in the glomeruli was associated with a significantly increased likelihood of response to immunosuppression.^107^ More disease-specific biomarkers, including levels of Gd-IgA1, anti–Gd-IgA1 antibodies, and IgA1-IgG immune complexes have been proposed to predict disease severity,^108,109^ but a significant overlap exists between levels in those with poor renal survival and in those with stable disease or healthy participants. The levels of circulating poly-IgA immune complexes, measured with a recombinant CD89 probe, were associated with clinical and pathologic markers of disease severity and decreased in response to immunosuppressive treatment.^110^

### Optimization of Supportive Care

**Figure 3:**
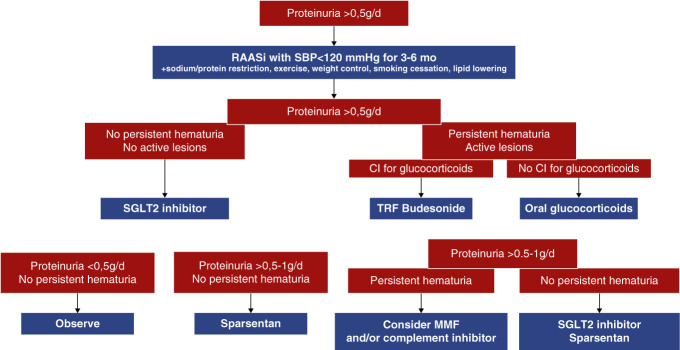
**Proposal for an individualized treatment approach in patients with IgA nephropathy.** CI, contraindication; MMF, mycophenolate mofetil; TRF, targeted-release formulation; SGLT2, sodium–glucose transporter 2.

IgA nephropathy is a CKD with slow but relentless nephron damage as a consequence of inflammation and fibrosis. As in any other CKD, supportive care aimed at reducing cardiovascular risk, unloading the glomerular pressure, and counteracting the tubular consequences of proteinuria remains the cornerstone of the therapy.^24,25^ Optimal supportive care consists of lifestyle modifications with smoking cessation, dietary sodium and protein restriction, weight control and exercise, statins in patients with hypercholesterolemia, BP control, and proteinuria reduction with maximally tolerated RAASi.^111^ Its value was epitomized by the observation that more than a third of patients who underwent optimization of supportive care and RAASi during the run-in phase of the STOP-IgA nephropathy trial had substantial reductions in proteinuria such that they were ineligible for subsequent randomization.^45^ The initial approach to all patients with IgA nephropathy (except special populations, see below) therefore consists of optimization of supportive care for at least 3 months, with the understanding that the 3-month period starts when target BP has been achieved (Figure 3). On the basis of the compelling evidence for their nephroprotective effects, SGLT2 inhibitors should be an integral part of contemporary optimization of supportive care, particularly in patients who do not qualify for immunosuppressive treatment or have residual proteinuria despite immunosuppression. The promising results of sparsentan suggest that it also merits inclusion in the algorithm of stepwise optimization of nonimmunologic treatment. However, its high economic cost calls for judicious use. In our opinion, sparsentan should be prioritized for those patients at high risk of disease progression, either because they have chronic lesions not amenable to immunosuppression or they have severe active disease that fails to go into rapid remission with immunosuppressive therapy (Figure 3). Optimization of RAASi to further reduce proteinuria by combining angiotensin-converting enzyme inhibitor and angiotensin II receptor blocker or adding a mineralocorticoid receptor antagonist is at the discretion of the treating physician. At every step of the algorithm, diuretics may be added to control persistent hypertension.

### Choice of Immunosuppressive Therapy

The Kidney Disease Improving Global Outcome guidelines suggest to start glucocorticoids with caution when proteinuria remains >1 g/d after at least 3 months of optimal supportive care.^102^ However, the use of proteinuria as sole criterium does not allow to discriminate between immunologically active disease and irreversible structural damage to the glomerular filtration barrier. As such, patients with extinguished disease and merely chronic scarring may be exposed to unnecessary glucocorticoid toxicity. In addition, early active disease with proteinuria <1 g/d may be denied the benefits of achieving remission with immunosuppression.

A personalized risk assessment should combine clinical and pathologic data^112^ (Figure 3). In patients with clinical risk factors for disease progression (proteinuria >0.5–1 g/d and persistent microscopic hematuria), the presence of predominantly active proliferative lesions (higher M and/or E scores), crescents (higher C score) or S1 lesions with podocytopathic features, is an indication for immunosuppressive therapy. Conversely, in case of advanced renal failure (eGFR chronically <30 ml/min per 1.73 m^2^) and isolated chronic lesions (typically >50% glomerulosclerosis and tubulointerstitial fibrosis), aggressive therapy should be withheld. However, many patients do not fall in those two extreme categories but have mixed active and chronic lesions. Therapy should be individualized in those cases, taking into account the evolution of clinical parameters and risk of side effects. For example, a patient with proteinuria 0.75 g/d, despite 6 months of maximal RAASi and SGLT2i, eGFR >50 ml/min, and MEST score M1E0S1T1C0 12 months ago, but persistent hematuria (>20 rbc/hpf), in our views, should be given a trial of immunosuppressive therapy.

A 6–9-month course of oral glucocorticoids remains the first-line immunosuppressive therapy in most patients. The moderate dose glucocorticoid regimen used in TESTING^46^ (0.4 mg/kg per day, maximum 32 mg/d, weaning by 4 mg/d per month) has demonstrated efficacy and relative safety and seems to be a good choice among the multiple available treatment regimens.^45,113–115^ In our experience, the Pozzi regimen that combines high-dose intravenous pulses with moderate-dose oral methylprednisolone also has a favorable toxicity profile. Pneumocystis prophylaxis should be added to mitigate the infection risk.

TRF-budesonide is a promising alternative to oral glucocorticoids, assuming equal or better efficacy and lower toxicity, although no direct comparisons are available. However, its high economic cost calls for restrictive use and careful cost–benefit considerations. We suggest to reserve it for patients with severe contraindications to oral glucocorticoids. MMF with or without low-dose steroids could also be proposed in patients with contraindications to high-dose steroids. The evidence in favor of complement inhibitors is still preliminary but very encouraging. We believe they should not be given as monotherapy but rather as adjunctive treatment to oral glucocorticoids or TRF-budesonide in patients with severe and active disease. Therapies directed at CD38, BAFF, and APRIL still have to prove value but may hopefully replace or complement broad immunosuppressants in the future. Although the evidence on hydroxychloroquine is scanty, it may be a good choice in patients with residual proteinuria after other treatment options have been exhausted.

The optimal timing of immunosuppression with respect to kidney biopsy remains moot. Unless there is evidence of progressive loss of kidney function, we advise to optimize supportive therapy for 3–6 months before starting immunosuppressive therapy. In the TESTING trial, the effects of glucocorticoid treatment were similar in patients treated within the first year of kidney biopsy or thereafter, suggesting that a delayed start may not be harmful. The chronic or relapsing-remitting nature of IgA nephropathy requires continuous monitoring beyond the initial treatment course. As discussed above, the beneficial effect of immunosuppressive agents (systemic glucocorticoids, MMF, TRF-budesonide) wanes after withdrawal of treatment. Repeated treatment cycles or maintenance therapy may therefore be required. In our experience, many patients showing evidence of a renal flare benefit from a short course of corticosteroids and adding MMF.

### Variant Forms

Rapidly progressive disease (defined as a ≥50% decline in eGFR over ≤3 months and >50% crescentic glomeruli on kidney biopsy) has a poor prognosis^116^ and qualifies for urgent treatment with glucocorticoids and cyclophosphamide.^102^ Staphylococcus-associated GN with dominant IgA staining should be ruled out in these cases.^117^ Early treatment with glucocorticoids is also recommended for patients with IgA nephropathy and minimal change-like lesions.^118^

## Conclusion

The main challenge in the approach to patients with IgA nephropathy is to estimate the degree of disease activity and the extent of preexisting chronic damage and predict the risk of renal function decline from either ongoing inflammation or progression of CKD. Subsequently, therapy should be individualized to target the factors judged to be most decisive for prognosis. Disease-specific treatment options are currently the subject of intense research, with promising preliminary results. However, before these novel therapies can supersede systemic immunosuppressants, well-designed cost-effectiveness analyses need to be undertaken, followed by a debate within the nephrologic community on how to prioritize these therapies.

## Data Availability

All data is included in the manuscript and/or supporting information.
